# "Il Faut Cultiver Notre Jardin"

**DOI:** 10.3201/eid1206.AC1206

**Published:** 2006-06

**Authors:** Polyxeni Potter

**Affiliations:** * Centers for Disease Control and Prevention, Atlanta, Georgia, USA

**Keywords:** Art and science, emerging infectious diseases, Albrecht Dürer, Self-Portrait with Sea Holly, weeds, thistle, malaria, HIV/AIDS

**Figure:**
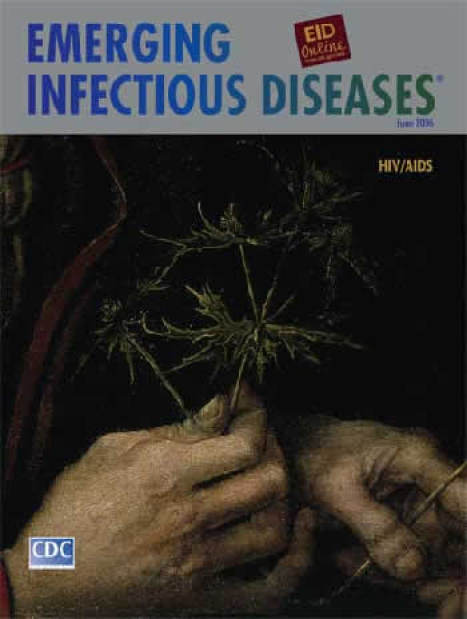
Albrecht Dürer (1471–1528). Self-Portrait with Sea-Holly (1493) Parchment mounted on canvas (56 cm × 44 cm). Photo: Arnaudet. Réunion des Musées Nationaux/Art Resource, NY. Louvre, Paris, France

—Voltaire^1^

"This I drew, using a mirror; it is my own likeness, in the year 1484, when I was still a child," wrote Albrecht Dürer about his first self-portrait, sketched with great confidence and skill when he was 13 years old (*1*). Fascinated by his own image, he continued to paint it throughout his life. His faithful dog once barked and wagged its tail at a newly completed self-portrait of his master, related his friend humanist scholar Konrad Celtis (1459–1508), repeating a story based on the anecdote from Roman writer Pliny (*1*).

Though born into the goldsmith trade on his father's side of the family, Dürer also apprenticed with painter and woodcut illustrator Michael Wolgemut, who introduced him to commercial bookmaking. At age 19, he left his native Nürnberg to wander the world and improve his skills, as was the custom after successful apprenticeships. In Basel, Strasbourg, Vienna, and Venice, he came to know and excel in diverse styles and techniques. A painter, as well as master printmaker and engraver, he was the first serious artist to work in watercolor. His landscapes, painted directly from nature, were matched in his day only by those of Leonardo da Vinci.

"The art of measurement being the foundation of all painting," he wrote, "I propose to give the elements thereof and to explain its principles to young people wishing to educate themselves in their art, so that they may confidently start measuring with a pair of compasses and ruler, thereby recognizing and having before their eyes the genuine truth…." (*2*). An artist working by the rule of thumb, without theoretical foundation, Dürer maintained, was "a wild, unpruned tree," in need of the objective and rational standards of the Renaissance (*3*).

These standards, acquired during his travels abroad, particularly Italy, and brought back to Germany, he also embraced in his personal life. A mathematician and humanist, he sought knowledge across diverse fields. An innovator and tradesman, he applied his artistic skills to new technologies and the production of high quality prints for the open market. His radical techniques caught the attention of the intellectual elite of his day. His closest friend and mentor was one of Nürnberg's most distinguished scholars, Willibald Pirckheimer, translator of Hellenic texts into Latin and German. Dürer's copper plate portrait of Pirckheimer, created from printed graphics, contained the inscription "Man lives through his intellect; all else will belong to death" (*4*).

"What beauty is I know not," Dürer pondered in his Book on Human Proportions. "In some things we consider that as beautiful which elsewhere would lack beauty." Beauty as the goal of art continued to excite his imagination and later featured in his treatises on measurement and fortification. His drawings after Andrea Mantegna and Antonio Pollaiuolo reflected his appreciation of Italian painting, which at its peak was guided by knowledge of geometry and proportion. Near the end of his life, Mantegna heard that Dürer was in Italy and sent for him, "in order to instruct Albrecht's facility and certainty of hand in his own understanding and skill" (*5*). But Mantegna died before Dürer could reach Mantua, "the saddest event in all my life" (*5*).

"There, where the yellow spot is located, and where I point my finger, there it hurts," Dürer wrote on a pen and watercolor self-portrait he sent to a physician for consultation (*6*). This half-length portrait, The Sick Dürer (1471–1528), likely described the painter's illness contracted during travel to the Netherlands in 1520. "In the third week after Easter I was seized by a hot fever, great weakness, nausea, and headache," he wrote in his diary, "And before, when I was in Zeeland, a strange sickness came over me, such as I have never heard of from any man, and I still have this sickness" (*7*). In between periods of good health, the fever periodically returned until his death at age 56.

Self-portrait with Sea Holly, on this month's cover, painted when Dürer was 22, is the earliest known free-standing self-portrait. Hands are difficult to paint from a mirror image. These (cover detail) are rough, a painter's hands, cracked and smudged. Goethe, when he saw a preliminary version of the painting, recognized that they held a sprig of sea holly, a thistle-like plant regarded an aphrodisiac, which appears in other Dürer works. The plant's German name means "fidelity of man," and its presence in this self-portrait could be alluding to the painter's engagement to be married around that time.

Dürer's devotion to knowledge as the means to beauty and truth guided his life. In illness, he enlisted his knowledge of the human body to describe the pain, anticipating pain mapping several centuries later. He grasped that knowledge advances human life, from understanding the nature of beauty to curtailing the scourge of disease.

"He looked like a bundle of dried straw," said Pirckheimer of his dying friend, supporting speculation today that Dürer had malaria, an ancient and continuing scourge (*8*). Newer scourges have now sprung, not the least of them HIV/AIDS, a "rough thistle." Despite substantial progress in prevention and control, emerging HIV/AIDS takes a global toll, particularly among the young, the old, and the underprivileged, "a weed" still requiring of us to "get on our hands and knees and begin clearing… away" (*9*).

## Footnotes

1 We must cultivate our garden. From Voltaire's Candide.
